# Acetaminophen Overdose and Self-Induced Ethanol Enema

**DOI:** 10.7759/cureus.113291

**Published:** 2026-07-24

**Authors:** Amisha Ghotra, Nina K Mangat, Bhesh R Karki, Yitzhak Twersky

**Affiliations:** 1 Department of Osteopathic Medicine, William Carey University College of Osteopathic Medicine, Hattiesburg, USA; 2 Department of Health Sciences, School of Nursing Studies, University of Miami, Coral Gables, USA; 3 Department of Internal Medicine, Downstate Medical Center, New York, USA; 4 Department of Gastroenterology, St. John’s Medical Group, Far Rockaway, USA

**Keywords:** colitis, ethanol enema, ethanol-induced colitis, rectal administration of alcohol, toxicology

## Abstract

Ethanol is most commonly consumed orally, although rare cases of intentional rectal administration have been described. We present the case of a 37-year-old man who presented to the emergency department following intentional rectal administration of ethanol and a concomitant acetaminophen overdose with extensive review of literature. This unusual presentation highlights the diagnostic and management challenges associated with concurrent toxic exposures and emphasizes the importance of early recognition and multidisciplinary care.

## Introduction

Ethanol is commonly consumed orally, and some rare reports describe the rectal administration of ethanol, which can lead to significant gastrointestinal and systemic toxicity. The first step of ethanol metabolism is by the liver through first-pass hepatic metabolism. Enemas allow for rapid mucosal absorption of alcohol, which bypasses this first step and leads to elevated systemic blood alcohol concentrations, which can cause toxicity [1]. The clinical presentation typically includes abdominal pain, hematochezia, and profuse diarrhea [1]. Endoscopies of these reported cases show evidence of proctitis or colitis [1]. Case reports describe findings mimicking ischemic colitis on CT imaging. Endoscopy typically shows chemical-induced colitis with mucosal erosions [1-3]. Most cases resolve with conservative management, but complications like hemorrhage, metabolic derangements, and death have been reported [4-6]. Ethanol-induced colitis is an uncommon but important etiology in differentiating acute colitis. To the best of our knowledge, after a review of the published literature, no previous case has described concurrent intentional acetaminophen overdose and rectal ethanol administration. Previously reported cases have involved isolated ethanol enemas without concomitant toxic ingestions. A literature search of PubMed and Google Scholar using the terms "ethanol enema," "alcohol enema," "rectal ethanol," "chemical colitis," and "acetaminophen overdose" identified published reports of ethanol enemas but no prior reports describing simultaneous acetaminophen overdose. We present the unique case of a suicide attempt involving both intentional acetaminophen overdose and self-administered ethanol enema.

## Case presentation

**Figure 1:**
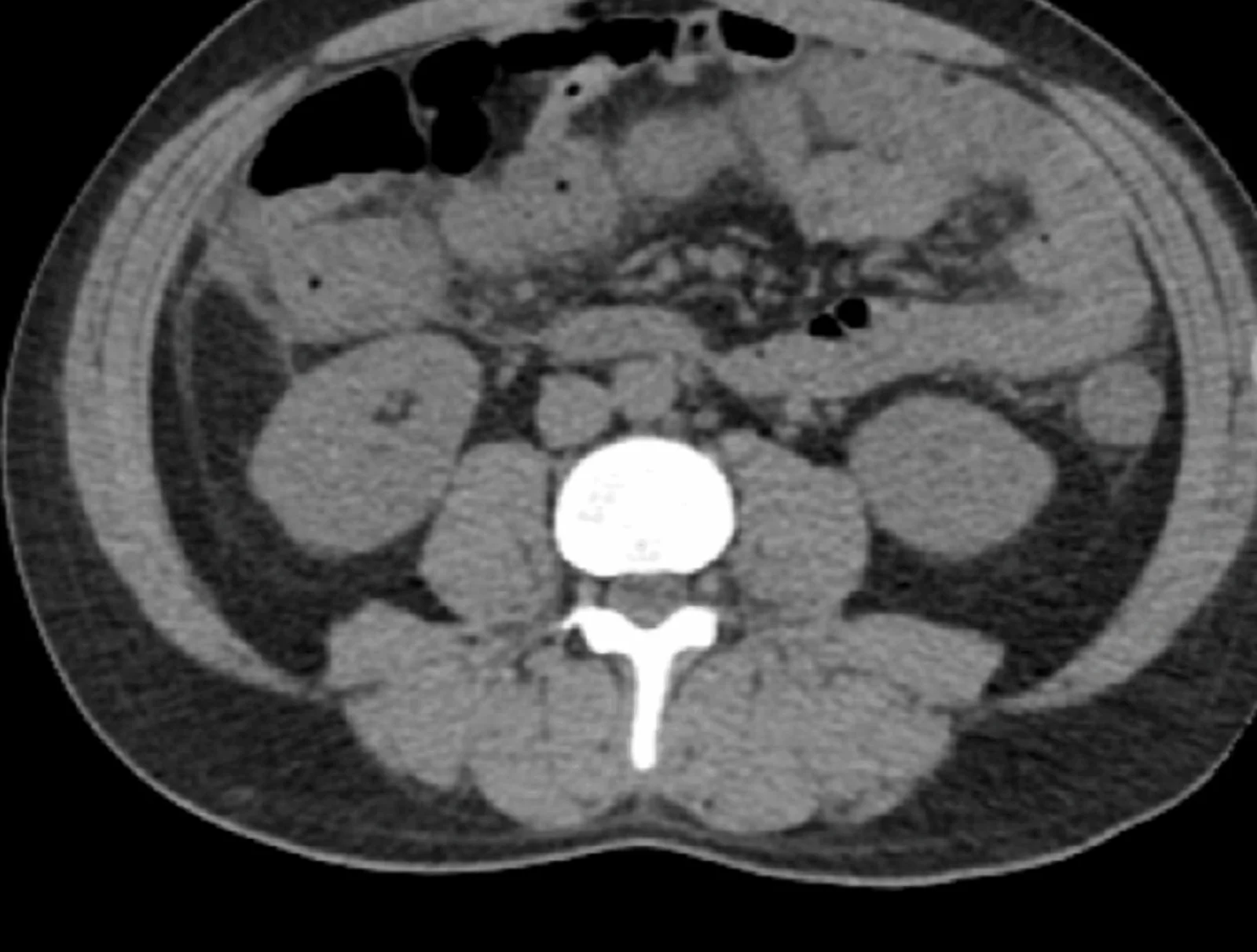
CT axial displaying colonic inflammation and fat stranding.

A 37-year-old man presented to the emergency department with a one-day history of diffuse and constant abdominal pain. He indicated that it was secondary to intentional acetaminophen overdose with the intent of suicide. He also reported nausea and vomiting, which began after the consumption of the acetaminophen. He reported taking 30 tablets of acetaminophen, approximately 16,000 mg, around 11 p.m. the night before arrival to the emergency department at 10 a.m. In the emergency department, the patient did not report any use of alcohol. The patient had a past medical history of rhabdomyolysis secondary to cocaine ingestion, major depressive disorder, and obsessive-compulsive disorder, with past hospitalizations due to psychiatric reasons, including attempted suicide, and chronic asymptomatic Hepatitis C infection. On physical exam, the patient’s vital signs were: temperature 98.9F, blood pressure (BP) 131/91, heart rate (HR) 71, peripheral oxygen saturation (SpO_2_) 98%. The patient was without any acute distress, alert, and oriented with normal mental status. Cardiac, respiratory, and neurological exams were normal. The gastrointestinal exam revealed a soft and nondistended abdomen with generalized abdominal tenderness, with no guarding or rebound. No jaundice or asterixis was noted. Labs are described in Table 1. CT abdomen revealed acute colitis of the transverse, sigmoid colon, and rectum (Figure 1).

**Table 1:** Serial laboratory values obtained throughout hospitalization. Initial evaluation demonstrated leukocytosis, mild hyperbilirubinemia, and elevated aspartate aminotransferase (AST) with a toxic serum acetaminophen concentration. Following treatment, the patient's acetaminophen level progressively declined to undetectable levels, liver function tests and bilirubin normalized, renal function remained preserved, and coagulation studies remained within normal limits. A reactive hepatitis C antibody was identified during hospitalization. Dashes (-) indicate laboratory values that were not obtained.

Laboratory Value	Day 0	Day 1	Day 3	Day 5	Day 7	Day 9	Reference Range
Hemoglobin (g/dL)	12.7	-	13.5	-	12.9	15.2	13.5-7.5
White Blood Cell Count (×10³/µL)	16.4	8.3	10.8	10.0	14.2	5.5	4.0-11.0
Platelet Count (×10³/µL)	269	-	325	318	311	389	150-400
Blood Urea Nitrogen (mg/dL)	16.0	13.0	4.0	5.0	6.0	11.0	7-20
Creatinine (mg/dL)	0.90	0.80	0.60	0.60	0.60	0.60	0.7-1.3
Total Bilirubin (mg/dL)	1.7	1.4	0.3	0.2	0.3	0.3	0.2-1.2
AST (U/L)	81	49	30	25	24	25	10-40
ALT (U/L)	28	23	22	23	24	21	7-56
Alkaline Phosphatase (U/L)	78	-	73	70	66	-	44-147
INR	1.15	-	0.92	-	-	1.21	0.8-1.2
Acetaminophen (µg/mL)	108	54	10	10	10	<10	<10
Hepatitis C Antibody	Not Obtained	Reactive	-	-	-	-	Nonreactive

**Figure 2:**
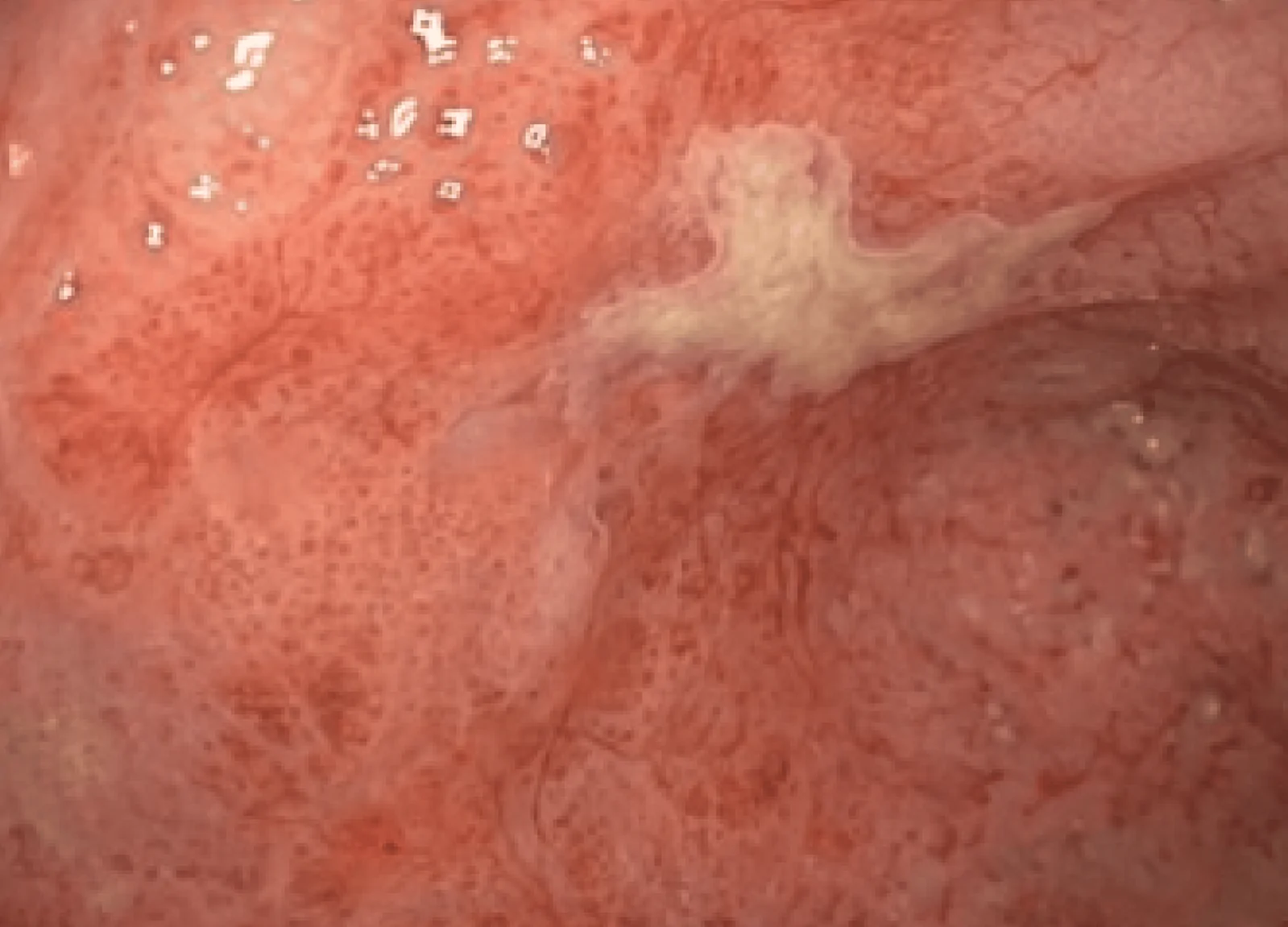
Colonoscopic images of mucosal inflammation of the rectum. Colonoscopy demonstrated continuous erythematous and friable mucosa with superficial erosions involving the rectum and sigmoid colon. No deep ulcerations or transmural necrosis were identified. Biopsies were not obtained because the endoscopic appearance was considered diagnostic for acute chemical injury and biopsy was unlikely to alter management.

The patient was admitted to inpatient medicine for an intentional acetaminophen overdose. Gastroenterology was consulted because of abnormal liver enzymes, concern for acetaminophen-associated hepatotoxicity, and subsequent hematochezia with CT evidence of colitis. Toxicology/Poison Control guided the management of acetaminophen overdose while Gastroenterology evaluated the gastrointestinal manifestations. Gastroenterology saw the patient at 3 p.m. the day of his hospital admission. At the time, his acetaminophen level was 108 (ug/mL), roughly 14 hours after ingestion. During interview of the patient by the GI team, the patient disclosed administering 1.5 L of vodka in his rectum the day prior before ingesting acetaminophen. He reported bloody, profuse, watery diarrhea that started immediately after the vodka administration. He had about six to eight loose bowel movements mixed with blood in total since the administration of the ethanol enema. After this disclosure, a colonoscopy was planned. Colonoscopy identified erythema and erosions in the rectal-sigmoid region, consistent with colitis (Figure 2). CT demonstrated inflammatory changes involving the transverse, sigmoid colon, and rectum, whereas colonoscopy identified the most prominent mucosal abnormalities within the rectosigmoid colon. This discrepancy likely reflects the greater sensitivity of CT for detecting mural edema over a broader segment of bowel, whereas endoscopy directly visualizes mucosal injury, which may be most pronounced distally following rectal ethanol administration. Stool infectious testing was not performed because the clinical presentation was highly consistent with chemical colitis following a toxic exposure.

The patient was initially admitted for acetaminophen overdose and started on N-acetyl cysteine (NAC) protocol [7]. After identifying the ethanol enema, the treatment plan was extended to include treatment for colitis. Poison Control was contacted immediately and recommended intravenous N-acetylcysteine therapy according to established acetaminophen overdose protocols. Intravenous N-acetylcysteine was initiated using the standard 21-hour protocol because the patient presented approximately 11 hours after ingestion with a toxic acetaminophen concentration of 108 μg/mL. Therapy was continued with serial monitoring of acetaminophen concentrations, liver function tests, and international normalized ratio (INR) until acetaminophen levels became undetectable and hepatic function remained stable. The patient was placed on 1:1 observation and was compliant with treatment. Antibiotics were not indicated. The patient’s liver enzymes continued to improve with a normal INR throughout his hospital stay. Transfer to a liver transplant center was initially discussed because of the potentially delayed evolution of acetaminophen-induced liver injury after a large intentional overdose. However, the patient's INR remained normal, bilirubin and transaminases improved, and he never met criteria for acute liver failure or transplant evaluation. For the patient's bloody diarrhea, intravenous fluid was administered, and oral intake was encouraged. Over the course of the hospital stay, the patient reported resolution of abdominal pain after five days and resolution of diarrhea after nine days. He was signed off the gastroenterology service and transferred to inpatient psychiatric care for two weeks. At psychiatric discharge, gastrointestinal symptoms had resolved.

**Table 2:** Clinical Timeline of Presentation, Management, and Hospital Course ED: Emergency department; NAC: N-acetyl cysteine.

Hospital Day	Event
Day -1	1.5 L vodka enema
Later	16 g acetaminophen ingestion
Day 0	ED presentation
Day 0	NAC initiated
Day 0	Poison Control consulted
Day 1	Colonoscopy
Day 5	Abdominal pain resolved
Day 9	Diarrhea resolved
Day 9	Transferred to psychiatry

## Discussion

Dual ingestions are exceedingly uncommon and present a unique set of challenges. For this case of dual ingestion, there was the risk of hepatic injury from the acetaminophen overdose, intoxication from ethanol, and direct injury to the colon from rectal administration of ethanol. Our patient was treated for both acetaminophen overdose and ethanol-induced colitis. The initial management was N-acetyl cysteine for the treatment of acetaminophen overdose to prevent hepatotoxicity [7]. Acetaminophen levels continued to decline after admission. Because the rectal ethanol ingestion was 11 hours before evaluation in the ED, he did demonstrate intoxication from ethanol. Colitis was managed conservatively. The patient’s laboratory abnormalities included white blood count (WBC) 16.4 (103 µg/mL), acetaminophen levels 108 µg/mL and aspartate aminotransferase (AST) 81 U/L. The elevated AST could either be explained by hepatic injury from the acetaminophen or ethanol. Mild AST elevation was most likely related to acute acetaminophen exposure, ethanol toxicity, or both. Although the patient had a reactive hepatitis C antibody, hepatitis C virus (HCV) RNA testing was unavailable; therefore, chronic active hepatitis could not be confirmed and should not be assumed to explain the transaminase elevation. Supportive management focused on intravenous fluid replacement for diarrhea-associated volume losses while serially monitoring hepatic and renal function.

Although ischemic, infectious, and inflammatory colitis were considered, the immediate onset of profuse bloody diarrhea following rectal ethanol administration strongly favored chemical colitis. CT imaging and colonoscopy demonstrated inflammatory mucosal injury without findings suggestive of transmural ischemia. The absence of a prior history of inflammatory bowel disease and the close temporal relationship with ethanol exposure made inflammatory bowel disease unlikely. Acetaminophen overdose rarely causes direct colonic injury and is therefore an unlikely explanation for the patient's lower gastrointestinal findings. A serum ethanol concentration was not obtained, limiting the assessment of systemic ethanol absorption. Nevertheless, the immediate onset of bloody diarrhea following rectal ethanol administration and characteristic endoscopic findings strongly supported chemical colitis.

Ethanol colitis is the inflammation of the colon after the introduction of ethanol into the colon. Colitis presents as diffuse abdominal pain, often accompanied by profuse watery diarrhea and blood, much like how our patient presented. The colonic mucosa is a site of rapid turnover, and ethanol has been shown to hinder the immunoregulatory process of the colonic mucosa along with the regenerative capacity [8]. Traditionally, we see colitis in the setting of inflammatory bowel disease, an immunological process, or in cases of ischemia or radiation. Ethanol administration has been a very rare cause of colitis. Primarily, ethanol-associated colitis cases have been seen in surgical preparation, where alcohol enemas are mixed with cleaning enemas [1]. Endoscopic findings of these patients have been consistent with those of our patient, displaying mucosal inflammation and erosions [8]. Metabolism of ethanol begins at the gastric mucosa, and the route of absorption plays a role in the first-pass metabolism of ethanol. Rectal absorption of ethanol is a mechanism that bypasses the first-pass metabolic effect by the stomach and liver; this allows for a higher concentration of blood ethanol, meaning a higher systemic concentration of ethanol [9].

Similar case reports of ethanol enemas do not have concomitant ingestions. A review of 12 reported alcohol enemas is listed in Table 2. The literature available shows that rectal alcohol administration largely involved distilled spirits, as compared to wine. Ten cases show ethanol enemas based on spirits or concentrated ethanol, and two involving wine-based enemas [1-12]. Mortality was not a common outcome, with only two reported deaths, both secondary to wine enemas [4,9]. Predominantly, the ethanol enemas resulted in inflammatory colitis, proctocolitis, or mucosal inflammation as described by six cases [3,6,10-13]. Ischemic injury was less frequent and only reported in two cases as ulcerations and a mimic of ischemic colitis [1,2].

**Table 3:** Review of Ethanol Enema Cases

Author (Year)	Age/ Sex	Type of Alcohol	Findings	Outcome	Other Overdose
Herrerias et al., 1983 [13]	NR	95% Ethanol	Proctocolitis	Recovered	No
Bhalotra, 1988 [12]	NR	Ethanol solution	Mucosal inflammation	Recovered	No
Triantafillidis et al., 1994 [6]	NR	95% ethanol	Inflammation	Recovered	No
Rodriguez-Tellez et al., 2001 [5]	NR	96% ethanol	Proctocolitis	Recovered	No
Michopoulos et al., 2000 [11]	NR	Alcohol	Clinicopathologic colitis	Recovered	No
Randolph et al., 2005 [2]	NR	Vodka	Mimicked ischemic colitis	Recovered	No
Mian et al., 2005 [1]	39 M	Vodka	Ulceration + ischemia	Recovered	No
Wilson et al., 2005 [4]	55 M	Wine	Not reported	Death	No
Pizzute, 2006 [10]	23 F	Vodka	Inflammation	Recovered	No
Andrade et al., 2003 [8]	Mice	50% ethanol	Colitis	Recovered	No
Rosendahl et al., 2018 [3]	NR	Distilled liquor	Diffuse inflammation	Recovered	No
Peterson et al., 2014 [9]	52 M	Sherry wine	Fatal ethanol intoxication	Death	No

## Conclusions

Colitis can be seen due to a myriad of etiologies, including infectious, inflammatory, radiation, ischemic, and chemical. Ethanol-induced colitis is rare and can lead to catastrophic complications, especially with concomitant overdose. Dual toxicologic processes can exacerbate the inflammatory response, leading to clinical complications for individuals using ethanol enemas. Supportive care remains the standard care, with monitoring for the development of any complications.
